# Implementing the human right to science in the context of health: introduction to the special issue

**DOI:** 10.1093/jlb/lsae018

**Published:** 2024-09-15

**Authors:** Bartha Maria Knoppers, Michael J S Beauvais

**Affiliations:** Distinguished James McGill Professor Emerita, McGill University, Montreal, Canada; Faculty of Law, University of Toronto, Toronto, Canada

Like its better known siblings, the human right to benefit from scientific progress (‘the human right to science’ or HRS) celebrated 75 years in 2023. Initially articulated in the first draft of the *American Declaration of the Rights and Duties of Man*,^1^ then shortly thereafter in the *Universal Declaration of Human Rights*^2^ (UDHR), then some years later in the *International Covenant on Economic, Cultural and Social Rights*^3^ (ICESCR) and other regional human rights instruments^4^, the human right to science has been a ‘sleeping beauty’^5^ – largely ignored by the international community, policy-makers, scientists, and citizens. All the while its siblings such as the right to the highest attainable standard of health, the right to an adequate standard of living, the right to freedom of opinion and expression, the right to equality before the law, and the very idea of human rights have been the backbone to social and political movements.^6^

A late bloomer, the human right to science’s time has arrived. In an era where scientific advancements risk disrupting the very complementarity between science and humanity^7^ and challenge what it means to be human,^8^ there has been increased interest in using international law and cooperation to ensure that scientific advancement happens on humanity’s terms. Comprising a ‘supercluster’ of four clusters of rights – the right to scientific progress, socially responsible science, participation in scientific progress, and benefit from scientific progress – the right is now fully awoken.^9^

Bodies such as UN Educational, Scientific and Cultural Organization (UNESCO), the UN Committee on Economic, Social and Cultural Rights, and the UN Special Rapporteur in the field of cultural rights have in recent years begun to elucidate the content of the HRS, as well as its relationship to other human rights.^10^ Non-governmental organizations such as the American Association for the Advancement of Science (AAAS), the Global Alliance for Genomics and Health (GA4GH), and Geneva Science and Diplomacy Anticipator (GESDA) have also used the HRS as a catalyst for policy-making and advocacy. We can now say that there is a robust interest among the international community, policy-makers, and scientists. Yet, citizen interest in and engagement with the right remains unclear.

The project to understand the potential and limits of the HRS is well under way. Much remains undetermined, especially with respect to the right’s relationship with other human rights and, in particular, children’s rights.^11^ Indeed, the emergence of new bioscientific advances, such as heritable human genome editing and artificial intelligence for research and clinical practice, pose specific issues that may help to better understand the human right to science. Ongoing armed conflicts and war in Gaza, Mali, Sudan, Ukraine, and Yemen and elsewhere also pose difficult questions about the duty to protect science beyond these conflicts' more readily evident humanitarian dimensions.^12^ For without individuals and groups engaged in scientific endeavors, there will be no science from which to derive benefits for all.

While the UN Committee on Economic, Social and Cultural Rights’ 2020 *General comment No. 25* has brought about some developments and clarity surrounding the right's content, more experience is needed to establish state practice and then guide further action.^13^ Specifying indicators and building up a history of reporting on them is needed for establishing standards against which state practice can be judged.^14^ Indeed, previous work from one of us shows that States Parties’ reports to the Committee vary considerably in what they see as measures to support the right’s realization.^15^ Consistency is needed if the right’s obligations are to be seen as legitimate and taken seriously.

This increased activity and interest in the human right to science poses issues and questions in the area of health. How do the duties the HRS imposes apply to specific technologies and contexts within the domain of human health? What is the interaction of the HRS with other international human rights and ethical norms? How does the right inform issues concerning access to vital diagnostics and therapeutics? What of the vulnerabilities of particular groups to be potentially either harmed or benefited by scientific advancements?^16^

## I. THE ARTICLES OF THIS SPECIAL ISSUE

We are pleased to present a series of papers that explore some of these issues. These papers were presented at a three-day workshop at the Brocher Foundation in Geneva, Switzerland with the support of GESDA at the end of 2022. The authors were invited to examine the intersection of the HRS, health, and other areas of law that play a key role in the production and translation of biomedical knowledge. Certain authors examine selected case studies where the HRS has either already contributed to policy-making or where emerging technologies in the health domain may help give shape to the HRS. Other participants presented works on the role of anticipation under the HRS, which have been published as a special issue of *The International Journal of Human Rights*.^17^

The articles form part of a tradition of using human rights to govern health-related research and its clinical applications and highlight the diverse perspectives a human-rights approach imposes. The indivisibility of human rights means that a health-related issue never singularly engages the human right to science. And so some amount of disagreement regarding how to balance competing concerns is inevitable.

Boggio examines anticipation within the context of international human rights.^18^ He argues that governments and sometimes scientists must engage in three different types of anticipation: beneficial anticipation, responsible anticipation, and participatory anticipation. He notes how this anticipation must be pluralistic and participatory: the collective imagination of stakeholders is needed to envisage potential futures for technologies. To this end, he identifies the importance of engaging non-technical stakeholders while also managing the politicization of science. He leaves us with a quadripartite research agenda regarding anticipation theory and practice.

Molnár-Gábor uses the human right to science to posit a novel interpretation of European data protection law for the governance of health data.^19^ This interpretation is grounded on the principle of loyalty and Member States’ obligations to realize the human right to science (and the right to the highest attainable standard of health) as either parties to the ICESCR or the UDHR via customary international law. Foregrounding the importance of data science to the generation and translation of biomedical knowledge sets up a lens to see how rules governing personal data processing can help or hinder the realization of the human right to science. By doing so, she reminds us of the delicate balancing act that a fundamental-rights approach to data protection imposes and invites us to reconsider the assessment of prospective risks and benefits in health data processing.

Dove proposes using the human right to science to buttress the public interest as a legal basis for using and disclosing confidential information for scientific research purposes.^20^ He suggests that such use and disclosure can be possible without individual consent if it can be shown to be in the overriding public interest and enable everyone to benefit from scientific advances. In doing so, he provides a framework for how the human right to science could expand when confidential information may be disclosed for research purposes, but emphasizes the need for transparency, participation and consideration of ethical and social aspects. He pushes us to think beyond international human rights and to consider how this normative system coexists with ethical and social considerations within certain domains.

Greely provocatively argues that the varied limitations of the human right to science make it unsuitable for regulating neuroscience and its applications.^21^ He notes the inherent tensions within the right: how do we secure freedom of scientific inquiry while avoiding the harms of science? Despite his doubts, he sees the greatest potential to be for the regulation of basic science and the right’s potential to underscore the potential value of research. Emphasizing the right’s potential for grounding procedures, he proposes ‘HRS impact statements’ as ways for opening up dialogue about how different regulatory choices affect the HRS while seizing upon the justiciability of the right for strategic litigation. He ultimately concludes that the most promising avenue for human rights approaches to regulating applications of neuroscience may be focusing on civil and political rights in the application of neuroscience.

Ho meanwhile looks to the participatory and collaborative potential of rights-based approaches to propose a new regulatory strategy for artificial intelligence-based medical devices (AIMDs).^22^ He posits that the unique innovation cycles of these technologies challenge conventional point-in-time regulation of devices. By focusing on the human right to science’s participatory dimensions, he argues that regulatory approaches should focus on collaboration and dialogue in the face of iterative improvements. He furthermore argues that regulatory networks that allow participation and a plurality of perspectives to emerge are an avenue to ensure that commercial actors alone do not set technological agendas and futures. Indeed, ensuring the right of everyone to benefit from science requires that ‘any interested person to participate and collaborate in shaping sociotechnical imaginations around adaptive AIMDs.’^23^

Van Beers, in a text on heritable human genome editing presented at the workshop, reminds us that scientific progress in the domain of health has a complex relationship with human rights.^24^ By focusing on the indivisibility of human rights – one cannot simply choose one above and beyond others – she argues that the development and application of science must serve goals compatible with the broader human rights framework. She notes the differences of approaches in using human rights frameworks in regulating heritable human genome editing. In this light, notions of safety and quality are not enough. She encourages us to ground interpretations of the human right to science in its status as a cultural right so that creativity and public debate come to the fore to decide the future application of heritable human genome editing.

Kunz, in a text on open science presented at the workshop, challenges assumptions that open science, the right to health, and HRS are complementary.^25^ She points out some of the inherent tensions of the HRS – guarantees of scientific freedom do not always sit easily with ensuring everyone benefits from scientific advancement. She encourages us to consider how law affects science and not necessarily in a positive way. If the international funding and regulation of science and scientific knowledge is solely based upon results, freedom of scientific inquiry can be at risk. The desire to ensure that science delivers benefits for humanity fosters an instrumental approach to science that may risk science’s autonomy.

## II. OPEN QUESTIONS

The contributions of this special issue of the *Journal of Law and the Biosciences* perhaps pose as many questions as they answer. Some of the most promising avenues for future scholarship relate to the conditions under which the HRS can be limited. The ICESCR requires that limitations to the rights within the Covenant, be ‘compatible with the nature of the rights and solely for the purpose of promoting the general welfare in a democratic society.’^26^ Which limitations are appropriate and for which aspects of the right?

The other side of the legitimacy of limitations concerns remedies for violations. The Committee underscores the importance of remedies at multiple points in its *General Comment 20*.^27^ If the human right to science is at the ‘vanishing point of economic, social and cultural rights’^28^, what can be said of the need for effective redress mechanisms and remedies? The limited use of the right before tribunals and committees makes the remedial question particularly difficult.^29^ Remedies may vary widely depending on whether the violation concerns a negative or positive obligation.^30^ From a remedial perspective, for example, ensuring non-discriminatory access to the benefits of scientific progress is quite different than removing barriers to scientific freedom.

This brings us to the context-specific nature of science. The breadth of activities covered by ‘science’ as a concept in international law is immense – from indigenous knowledge generation to the development of gene-based therapeutics by biotech companies to citizen-led data collection concerning the impact of climate change on their communities.^31^ Each of these scientific endeavors implicates different communities, different concerns, and different issues. While a human rights-based approach may provide a common framework for discussing and thinking through issues, further research must continue to develop a necessary pluralism of sciences and of contexts.

The connections between Open Science and the human right to science are evident. Both emphasize broad participation and modifying hierarchies in the production and application of science. There is some literature on the interface of the access dimension of Open Science and the international cooperative dimensions of the human right to science.^32^ Yet greater attention is needed on science diplomacy and international cooperation to foster scientific advancement and the sharing of its benefits.^33^ What are the roles of scientific organizations in realizing the right to science? In which ways can the right to science reinforce mutual cooperation and promote the sharing of knowledge and scientific resources between the Global South and North?

Relatedly, the interface of intellectual property rights and the human right to science remains an important area of research and policy-making.^34^ The HRS includes the right to the protection of moral and material interests for scientific production,^35^ which is at least meant to be distinct from the protection intellectual property rights confer.^36^ The CESCR has already noted how certain approaches to intellectual property rights with respect to private scientific research can create distortions in the funding of scientific research, restrictions on sharing scientific knowledge and data, and limiting access to health products and technologies.^37^ With specific regard to health, the deep involvement of private-sector actors in the production and translation of biomedical knowledge poses challenging questions such as: What are the obligations to promote access and participation in scientific progress that impacts human health? How do the rights to science and health relate to intellectual property protection in international trade law?^38^

Beyond intellectual property, the road of progress is likewise full of challenges. For example, AI-based interventions are beginning to change healthcare. Their potential for harm requires that they be integrated within a normative system such as the international human rights order. Of course international human rights cannot provide us with all the answers,^39^ but they provide legitimate building blocks from which law and policy can be built to ensure alignment with human values.^40^ In this way, the powers and promise of artificial intelligence can be oriented and regulated to support, not threaten, this ambitious road to human health.

What of the HRS, social and political movements, and research projects? The right to health has served as an important, albeit imperfect, foundation for obligations with respect to access to medicines.^41^ By focusing on the right of ‘everyone to benefit from scientific progress’, the very text of the right may ground claims to participate and benefit from science, including ethical deliberation on science. The calls for greater diversity and representation in scientific data dovetail with the participatory dimensions of the HRS. Endeavors such as the NIH’s All of Us, the Human Pan-Genome Reference Map, and the Human Cell Atlas aim to ensure that the health sciences are more inclusive and benefit a broader spectrum of the population.

This brings us to questions of scientific diplomacy and policy-making. The CESCR and Besson have each underscored the importance of institutions and cooperation for science.^42^ The COVID-19 pandemic has further highlighted the importance of citizenship and solidarity.^43^ With the WHO currently drafting an instrument on pandemic prevention, preparedness and response, data sharing obligations and institutional supports can be partially grounded in the HRS while being balanced with other rights and obligations.^44^ Indeed, turning to international human rights imports considerations of non-discrimination, equity, transparency, participation, and other essential elements to trustworthy governance.

Science policy, whatever its potential tensions with science itself, must emphasize the right of all to participate and benefit from science. In the domain of human health, securing beneficial outcomes for current and future patients requires robust institutions, cooperation, and acceptable partnerships between the public and private sectors that result in better health outcomes for humanity.^45^ Bridging the ever-larger gaps between wealthy countries and low- and middle-income countries should be a priority. As the former begins to realize precision medicine, the latter two still lack adequate access to many essential medicines for common diseases.

There remains much work to be done. Prospective policy-building may well seek to achieve the ‘right to benefit’ by building on lessons learned. But implementation is the true test. One hopes that this special issue will modestly contribute to that effort.

## Footnotes

1 Inter-American Commission on Human Rights (IACHR), American Declaration of the Rights and Duties of Man (1948).

2 United Nations General Assembly, Universal Declaration of Human Rights, 217 A (III) 27 (1948).

3
United Nations General Assembly, International Covenant on Economic, Social and Cultural Rights, United Nations, *Treaty Series*, vol. 993, p. 3 (1966).

4
Cesare P.R. Romano & Andrea Boggio, Right to Science, Max Planck Encyclopedia of Comparative Constitutional Law (Rainer Grote et al. eds., 2020), https://oxcon.ouplaw.com/display/10.1093/law-mpeccol/law-mpeccol-e831 (last visited Aug 25, 2023).

5 William A. Schabas, *Looking Back : How the Founders Considered Science and Progress in Their Relation to Human Rights*, 2015 European Journal of Human Rights 16.

6 See generally Neil Stammers, *Social Movements and the Social Construction of Human Rights*, 21 Human Rights Quarterly 980 (1999); Aryeh Neier, The International Human Rights Movement: A History (2020).

7
Helmuth Plessner, Levels of Organic Life and the Human: An Introduction to Philosophical Anthropology (J. M. Bernstein tran., 2019) cited in Britta van Beers, *Heritable human genome editing and the right to science: The case for a holistic and inclusive approach*(2022). On file with authors.

8 Bartha Maria Knoppers & Henry T. Greely, *Biotechnologies Nibbling at the Legal ‘Human*,’ 366 Science 1455 (2019).

9
Cesare P. R. Romano & Andrea Boggio, The Human Right to Science: History, Development, and Normative Content (2024).

10 United Nations General Assembly, Declaration on the Use of Scientific and Technological Progress in the Interests of Peace and for the Benefit of Mankind (1975); UN Educational, Scientific and Cultural Organization (UNESCO), Declaration on Science and the Use of Scientific Knowledge (1991); UN Educational, Scientific and Cultural Organization (UNESCO), Universal Declaration on the Human Genome and Human Rights (1997); UN Educational, Scientific and Cultural Organization (UNESCO), Recommendation on Science and Scientific Researchers (2017); United Nations Committee on Economic, Social and Cultural Rights, General Comment No. 25 (2020) on Science and Economic, Social and Cultural Rights (Article 15 (1) (b), (2), (3) and (4) of the International Covenant on Economic, Social and Cultural Rights) (2020); United Nations Educational, Scientific and Cultural Organization (UNESCO), UNESCO Recommendation on Open Science (2021).

11 See also Michael D.A. Freeman, A Magna Carta for Children?: Rethinking Children’s Rights (2020).

12 International Science Council, *Protecting Science in Times of Crisis* (2024), https://council.science/publications/protecting-science-in-times-of-crisis/ (last visited Apr 30, 2024).

13 Andrew Mazibrada, Monika Plozza & Sebastian Porsdam Mann, *Innovating in Uncharted Terrain: On Interpretation and Normative Legitimacy in the CESCR’s General Comment No. 25 on the Right to Science*, 28 The International Journal of Human Rights 148 (2023).

14 Andrea Boggio, *Anticipation in the Biosciences and the Human Right to Science*, 11 Journal of Law and the Biosciences lsae002 (2024).

15 Rumiana Yotova & Bartha M. Knoppers, *The Right to Benefit from Science and Its Implications for Genomic Data Sharing*, 31 Eur J Int Law 665 (2020).

16 Audrey R. Chapman, *Towards an Understanding of the Right to Enjoy the Benefits of Scientific Progress and Its Applications*, 8 Journal of Human Rights 1 (2009).

17 See The International Journal of Human Rights 28(3) (2024). https://www.tandfonline.com/toc/fjhr20/28/3.

18 Boggio, *supra* note 14.

19 Fruzsina Molnár-Gábor, *Implementing the Human Right to Science in the Context of Health-Related Data Processing*, 11 Journal of Law and the Biosciences lsae004 (2024).

20 Edward S. Dove, *Confidentiality, Public Interest, and the Human Right to Science: When Can Confidential Information Be Used for the Benefit of the Wider Community?* 10 Journal of Law and the Biosciences lsad013 (2023).

21 Henry T. Greely, *Implementing the Human Right to Science in Neuroscience*, 11 Journal of Law and the Biosciences lsae011 (2024).

22 Calvin W L Ho, *Implementing the Human Right to Science in the Regulatory Governance of Artificial Intelligence in Healthcare*, 10 Journal of Law and the Biosciences lsad026 (2023).

23
*Id.* at 30.

24 van Beers, *supra* note 7.

25 See Raffaela Kunz, *The Rights to Health and Science and Open Science – Friends or Foes?* (2022). On file with authors.

26 United Nations General Assembly, *supra* note 3 at 4; United Nations Committee on Economic, Social and Cultural Rights, *supra* note 10 at 50.

27 United Nations Committee on Economic, Social and Cultural Rights, *supra* note 10 at 45, 52, 84, and 89; United Nations Committee on Economic, Social and Cultural Rights, General Comment No. 17: The Right of Everyone to Benefit from the Protection of the Moral and Material Interests Resulting from Any Scientific, Literary or Artistic Production of Which He or She Is the Author (Art. 15, Para. 1 (c) of the Covenant), 51–54 (2006).

28
William A. Schabas, *Study of the Right to Enjoy the Benefits of Scientific and Technological Progress and Its Application*, in Human rights in education, science, and culture: legal developments and challenges 273, 274 (Yvonne Donders & Vladimir Volodin eds., 2007).

29 Artavia Murillo, et al. (‘In Vitro Fertilization’) v. Costa Rica, (2012) (Inter-American Court of Human Rights); S. C. and G. P. v. Italy, (2019) (Committee on Economic, Social and Cultural Rights).

30 See also Yvonne Donders, *The Right to Enjoy the Benefits of Scientific Progress: In Search of State Obligations in Relation to Health*, 14 Medicine, Health Care, and Philosophy 371 (2011), who discusses the unique aspects of the HRS from a remedial perspective.

31 See the definition of ‘science’ in United Nations Educational, Scientific and Cultural Organization (UNESCO), *supra* note 10. A modified version of this definition, which requires falsifiability, was adopted in United Nations Committee on Economic, Social and Cultural Rights, *supra* note 10. For a criticism of this choice see Mazibrada, Plozza, and Porsdam Mann, *supra* note 13.

32 Raffaela Kunz, *Opening Access, Closing the Knowledge Gap? Analysing GC No. 25 on the Right to Science and Its Implications for the Global Science System in the Digital Age*, 81 Zeitschrift für ausländisches öffentliches Recht und Völkerrecht / Heidelberg Journal of International Law 23 (2021).

33 United Nations Committee on Economic, Social and Cultural Rights, *supra* note 10 at 77. See also Samantha Besson, Quel droit et quelles institutions pour une « république universelle des sciences » ?, (2024), https://www.college-de-france.fr/fr/agenda/cours/le-droit-international-de-la-science/quel-droit-et-quelles-institutions-pour-une-republique-universelle-des-sciences.

34 Aurora Plomer, *The Human Rights Paradox: Intellectual Property Rights and Rights of Access to Science*, 35 Human Rights Quarterly 143 (2013); Aurora Plomer, *IP Rights and Human Rights: What History Tells Us and Why It Matters*, in The Right to Science: Then and Now 54 (Helle Porsdam & Sebastian Porsdam Mann eds., 2021); Klaus D. Beiter, *Establishing Conformity Between TRIPS and Human Rights: Hierarchy in International Law, Human Rights Obligations of the WTO and Extraterritorial State Obligations Under the International Covenant on Economic, Social and Cultural Rights*, in TRIPS plus 20: From Trade Rules to Market Principles 445 (Hanns Ullrich et al. eds., 2016).

35 United Nations General Assembly, *supra* note 2 at 27(2); United Nations General Assembly, *supra* note 3 at 15(1)(c).

36 This aspect of the HRS is meant to protect a link between one’s contributions to scientific (and other cultural) outputs and the potential to enjoy an adequate standard of living. See United Nations Committee on Economic, Social and Cultural Rights, *supra* note 27; Plomer, *supra* note 34.

37 United Nations Committee on Economic, Social and Cultural Rights, *supra* note 10 at 61.

38
Ping Xiong, An International Law Perspective on the Protection of Human Rights in the TRIPS Agreement: An Interpretation of the TRIPS Agreement in Relation to the Right to Health (2012).

39 John Tasioulas, *Conceptual Overreach Threatens the Quality of Public Reason*, Aeon (2021), https://aeon.co/essays/conceptual-overreach-threatens-the-quality-of-public-reason (last visited May 3, 2024).

40
Vincent C. Müller, *Ethics of Artificial Intelligence and Robotics*, *in*The Stanford Encyclopedia of Philosophy (Edward N. Zalta & Uri Nodelman eds., 2020), https://plato.stanford.edu/archives/fall2023/entries/ethics-ai/ (last visited May 3, 2024).

41 S. Katrina Perehudoff, Nikita V. Alexandrov & Hans V. Hogerzeil, *The Right to Health as the Basis for Universal Health Coverage: A Cross-National Analysis of National Medicines Policies of 71 Countries*, 14 PLOS One e0215577 (2019).

42 United Nations Committee on Economic, Social and Cultural Rights, *supra* note 10; Besson, *supra* note 33.

43 International Bioethics Committee & World Commission on the Ethics of Scientific Knowledge and Technology, *Statement on COVID-19: Ethical Considerations from a Global Perspective*, (2020), https://unesdoc.unesco.org/ark:/48223/pf0000373115; Yuval Noah Harari, *The World after Coronavirus*, The Financial Times, Mar. 20, 2020.

44 Intergovernmental Negotiating Body (INB), https://inb.who.int (last visited May 3, 2024).

45
Lincoln C. Chen, Tim G. Evans & Richard A. Cash, Health as a Global Public Good, in Global Public Goods: International Cooperation in the 21st Century 284 (Inge Kaul, Isabelle Grunberg, & Marc Stern eds., 1999).

